# Herbivory and anti-herbivore defences in wild and cultivated *Cnidoscolus aconitifolius*: disentangling domestication and environmental effects

**DOI:** 10.1093/aobpla/plaa023

**Published:** 2020-06-06

**Authors:** Virginia Solís-Montero, Daniela A Martínez-Natarén, Víctor Parra-Tabla, Carlos Ibarra-Cerdeña, Miguel A Munguía-Rosas

**Affiliations:** 1 Centro de Investigación y de Estudios Avanzados del Instituto Politécnico Nacional (Cinvestav), Merida C.P., México; 2 Consejo Nacional de Ciencia y Tecnología (Conacyt), Insurgentes Sur, Ciudad de México C.P., México; 3 Departamento de Ecología Tropical, Universidad Autónoma de Yucatán, Merida C.P., México

**Keywords:** Chaya, *Cnidoscolus aconitifolius*, crop wild relatives, domestication, herbivory, phenotypic plasticity, plant defence

## Abstract

Phenotypic changes in plants during domestication may disrupt plant–herbivore interactions. Because wild and cultivated plants have different habitats and some anti-herbivore defences exhibit some plasticity, their defences may be also influenced by the environment. Our goal was to assess the effects of domestication and the environment on herbivory and some anti-herbivore defences in chaya (*Cnidoscolus aconitifolius*) in its centre of domestication. Herbivores, herbivory, and direct and indirect anti-herbivore defences were assessed in wild and cultivated plants. The same variables were measured in the field and in a common garden to assess environmental effects. Our results show that domestication increased herbivory and herbivore abundance, but reduced direct and some indirect defences (ants). The environment also affected the herbivore guild (herbivore abundance and richness) and some direct and indirect defences (trichome number and ants). There was also an interaction effect of domestication and the environment on the number of trichomes. We conclude that domestication and the environment influence herbivory and anti-herbivore defences in an additive and interactive manner in chaya.

## Teaser

Chaya (*Cnidoscolus aconitifolius*) is a crop species domesticated by the Maya in Mexico. In its domestication centre, chaya coexists with its wild relatives; however, domesticated chaya is grown in home gardens while wild chaya grows in nearby disturbed vegetation. Wild chaya is evidently thornier than cultivated, a trait that has been selected by growers over several years. In our study we found that domesticated plants are less defended and more attacked by herbivores and also that the environment prevailing in its habitat affects some of these defences.

## Introduction

Domestication is the outcome of artificial selection that leads to the increased co-evolutionary adaptation of plants to cultivation and their utilization by humans (Gepts 2010). However, the independent selection of traits relevant to humans is unlikely and often, some undesirable correlated traits are unconsciously selected during the domestication process (Chen *et al.* 2015; Whitehead *et al.* 2017) or there may be trade-offs between different functions (e.g. growth vs. defence) (Milla *et al.* 2015; Züst and Agrawal 2017). An illustrative example is the reduction of anti-herbivory defences exhibited by some domesticated plants relative to their wild progenitors (e.g. Gaillard *et al.* 2018; Hernández-Cumplido *et al.* 2018).

In a recent meta-analysis, Whitehead *et al.* (2017) found that, though significant, the effect of domestication on herbivore resistance was highly variable among and within crops and, unexpectedly, they found that their analysis did not support the idea that domestication reduces herbivore resistance via a reduction of defences (i.e. plant domestication-reduced defence hypothesis; Chaudhary 2013; Hernández-Cumplido *et al.* 2018). The authors suggested that this was probably because several of the studies they revised were not specifically designed to test for the effect of domestication on herbivore resistance or defence and, in some cases, wild and cultivated plants were exposed to completely different environments (Whitehead *et al.* 2017). The latter should be controlled to separate the effect of domestication *per se* from environmentally induced variation. Wild and cultivated plants usually grow in different habitats. Because some plant traits associated with herbivore resistance are plastic (Abdala-Roberts and Parra-Tabla 2005; Heil 2010; Yamawo *et al.* 2012), some of the phenotypic differences exhibited between crops and their wild relatives may be of environmental origin. However, the extent to which the environment contributes to phenotypic divergence between crops and their wild progenitors is unknown (Gremillion and Piperno 2009). Typically, domesticated plants grow in environments that are richer in nutrients and water, and subject to less disturbance and less competition than their wild relatives are (Chen *et al.* 2015; Milla *et al.* 2015). The favourable conditions prevailing in the habitat of domesticated plants may favour the expression of phenotypes with a low level of defence (Folgarait and Davidson 1995; Yamawo *et al.* 2012). However, the effects of domestication *per se* and environmentally induced variation on herbivore resistance and anti-herbivore defence are frequently confounded in field studies (e.g. Mondolot *et al.* 2008; Turcotte *et al.* 2014; Chacón-Fuentes *et al.* 2015). Therefore, an approach that combines field data and common garden experiments is useful for separating domestication *per se* from environmental effects.

Most studies addressing the effect of domestication on herbivore resistance and defence have focused on direct defences (see supplementary material in Whitehead *et al.* 2017). However, indirect defences, which attract the natural enemies of the herbivores and reduce plant damage (Dicke *et al.* 2003), have received less attention (Chen *et al.* 2015). Domestication modifies some physical, nutritional and chemical traits that play an important role in herbivore location and attack by their natural enemies (Price *et al.* 1980; Cortesero *et al.* 2000) and this may disturb the capacity of natural enemies to regulate herbivore attack (Chen and Walter 2005; Macfadyen and Bohan 2010). However, for some groups of natural enemies such as parasitoids, no generalizable effect has been identified because the plant traits selected during domestication affect this interaction in opposite directions; i.e. some traits favour (e.g. less toxic compound in plants and their herbivores; Ode 2006) and others reduce (e.g. reduced volatile emissions; Chen *et al.* 2015) the regulatory activity of parasitoids. Ants that visit extrafloral nectaries are another group of natural enemies that plays an important defensive role (Rico-Gray and Oliveira 2007); however, it is currently unknown how they are affected by plant domestication. Indirect defences can also be affected by environmental factors. For example, a suitable host for some parasitoids may be unavailable in agroecosystems (Rodríguez-Saona *et al.* 2011; Chen *et al.* 2013) and the nutrient-rich conditions that prevail in agroecosystems may favour the production of extrafloral nectar and as a result, attract more ants (Yomawo *et al.* 2012).

Most domestication events originated within the native distribution range of wild ancestors (Meyer *et al.* 2012). In these areas, native herbivores and their natural enemies have a coevolutionary history, first with wild progenitors and then, with domesticated landraces (Chen *et al.* 2015, 2017). Therefore, examining the effects of plant domestication on plant–herbivore interactions in the centre of domestication offers an excellent model for assessing how novel phenotypes shaped by domestication affect herbivore resistance while controlling for the geographic and evolutionary history of endemic herbivores with crops and their wild progenitors (Chen *et al.* 2017). This is important because when crops are taken to new environments, they often encounter ecological conditions different from those experienced by their wild relatives, interact with new species of herbivores and cultivation practices (Chen *et al.* 2017).

Here, we looked at the effects of domestication *per se* and of the environment on herbivory, and direct and indirect defences in chaya (*Cnidoscolus aconitifolius*) in its domestication centre (Ross-Ibarra and Molina-Cruz 2002). In this area, cultivated plants are grown exclusively in home gardens (where wild plants are removed), while wild plants grow in disturbed secondary vegetation (Ross-Ibarra 2003; Munguía-Rosas *et al.* 2019). Therefore, under field conditions, the effect of domestication *per se* is confounded with environmental effects. Thus, to separate these effects, in addition to field observations, we simultaneously recorded herbivory and defences in a common garden. We hypothesized that domestication *per se* interacts with the environment and affect both herbivory and direct and indirect defences in chaya. Our predictions were: (i) domestication increases herbivory and reduces defences, and (ii) if environmental effects are important, differences between cultivated and wild plants in terms of herbivory and defences will be lower in the common garden than in their original habitats in the field

## Methods

### Study species

Chaya (*C. aconitifolius*: Euphorbiaceae) is a perennial shrub, up to 4.5 m tall (Standley and Steyermark 1949). In the study area, the flowering season extends from May to November with a flowering peak in summer (June–September). Fruit and pollen production is abundant in the wild but infrequent in cultivated plants (Ross-Ibarra 2003). The area of the leaf lamina of wild plants (151.52 ± 5.17 cm^2^; hereafter, mean values ± 1 SE) is significantly larger than that of cultivated plants (123.66 ± 5.17 cm^2^) (Solís-Montero 2019). Chaya exhibits urticant trichomes on stems, leaves and fruits (Parra-Tabla *et al.* 2004); wild plants appear to have more trichomes than cultivated plants do (Ross-Ibarra 2003). Some leaf traits (e.g. trichome number) exhibit phenotypic plasticity in chaya (Parra-Tabla *et al.* 2004; Abdala-Roberts and Parra-Tabla 2005). The main chewing herbivores are generalist caterpillars and grasshoppers for both wild (Parra-Tabla *et al.* 2004) and cultivated plants (Solis-Montero 2019). There are two extrafloral nectaries at the leaf base (Standley and Steyermark 1949). These are active during the day and are actively visited by ants that defend the plants from herbivores (M. A. Munguía-Rosas, unpubl. data). Hydrocyanic glycosides are present in the leaves of wild and cultivated plants (Kuti and Kuti 1999). Also, the content of condensed tannins in leaves was very similar in wild (1.34 ± 0.21 %) and cultivated plants (1.58 ± 0.18 %) (*F*_1, 16_ = 0.56, *P* = 0.51) in a subsample of the study plants (*n* = 20) collected during the study (M. A. Munguía-Rosas, unpubl. data).

Cultivation in the study area is usually from stem cuttings in home gardens (Munguía-Rosas *et al.* 2019). Wild plants reproduce sexually; however, asexual reproduction is also possible with human assistance. According to Ross-Ibarra (2003), chaya is a domesticated plant and its domestication centre is the Yucatan Peninsula. People in this area consume the boiled leaves in several dishes. Selection has focused on leaf palatability and size, i.e. bigger, softer leaves with fewer trichomes are preferred (Munguía-Rosas *et al.* 2019). Only one variety of the cultivated plant has been formally identified on the Yucatan Peninsula (Munguía-Rosas *et al.* 2019).

### Study area

Data were collected from two different environments: in the field and in a common garden, both located in the municipality of Merida on the Yucatan Peninsula (20°49′15″–21°01′18″N; 89°41′30″–89°33′18″W; 10–30 m a.s.l.). The field location is the habitat of both wild (disturbed secondary forest) and cultivated plants (home gardens). The secondary forest is dominated by the trees: *Leucaena leucocephala*, *Lysiloma latisiliquum*, *Piscidia piscipula* (Fabaceae), *Bursera simaruba* (Burseraceae) and the shrubs: *Gymnopodium floribundum* (Polygonaceae) and *Permentiera millspaughiana* (Bignoniaceae). In contrast, cultivated species such as papaya (*Carica papaya*), orange (*Citrus sinesis* and *C. aurantium*), lemon (*Citrus limon*) and cultivated chaya are the dominant species in home gardens. The common garden was a monoculture of chaya of ca. 2000 m^2^, established in the study area as part of a bigger project 2 years prior to this study. The garden had 67 plants, 33 wild and 34 cultivated, randomly located, from 1 to 1.5 m apart. Both wild and cultivated plants in the common garden were reproduced from stems obtained from the surroundings of the field and common garden locations. Mother plants were selected to be as similar as possible. No pesticides or fertilizers were used in the field locations or in the common garden during the study. The study area offers ideal conditions for conducting this research for several reasons. The most relevant are that (i) chaya was domesticated in the study area (Ross-Ibarra 2003), (ii) to this day chaya is cultivated by the same ethnic group that domesticated it and (iii) both cultivated and wild plants grow in close proximity.

The field and common garden locations did not differ significantly in relative humidity (*F*_2, 48_ = 2.78, *P* = 0.07). However, there were statistically significant differences for temperature (*F*_2, 48_ = 6.88, *P* < 0.01) and photosynthetically active radiation (*F*_2, 48_ = 4.45, *P* = 0.02). Home gardens had the lowest values (29.87 ± 0.11 °C; 257.21 ± 10.31 PPFD), the common garden had the greatest values (33.27 ± 0.11 °C; 665.21 ± 13.31 photosynthetic photon flux density [PPFD]) and in the secondary forest values were intermediate (31.72 ± 0.16 °C; 298 ± 67 PPFD). There were important differences among environments in terms of plant community composition (see above) and management. Plants in the home gardens were watered and undesirable wild plants removed, while no management was carried out in the secondary forest. In contrast to field locations, the common garden was a monoculture and as a result, environmental heterogeneity was limited relative to that of the field locations. While wild and cultivated plants inhabit different environments in the field, in the common garden, wild and cultivated plants shared the same environmental conditions. Therefore, with this experimental design, the effect of the environment (in a broad sense) can be inferred from the field versus common garden comparison, i.e. if herbivory and defences are very similar between plants in the field and the common garden, the environmental effect is negligible.

### Herbivores and herbivory

During the summer and fall of 2018, all herbivores observed from 0800 to 1200 h on wild and cultivated plants (*n* = 66) in both environments (in the field and in the common garden) were recorded. In the field, 20 wild and 20 cultivated plants were surveyed, while 13 wild and 13 cultivated plants were surveyed in the common garden. Fewer plants were surveyed in search of herbivores in the common garden than in the field because the former lost their leaves earlier in the season. Wild and cultivated plants were easily distinguished in the field because the former are evidently thornier and cultivated plants do not occur outside of the home gardens. To control for size and age (life stage), we selected 1.5–2 m tall, reproductive plants (i.e. plants producing flowers) in both environments. Each day, only one randomly selected wild plant and the cultivated plant nearest to it were sampled to minimize temporal and spatial variability (mean distance between each pair of plants = 600 ± 500 m). Herbivore sampling was conducted in different environments on alternate days. Only one cultivated plant per home garden was selected in the field, and contiguous home gardens were avoided to gain independence (the shortest distance between plants was 300 m). For each plant, the herbivory survey takes 1–2 h; therefore, only two plants could be surveyed during the predefined period (0800–1200 h). After 1200 h, herbivore activity decreases, and the owners of the gardens are less available (i.e. lunch and nap time).

Also, a random sample of three fully expanded leaves from 40 plants (20 wild and 20 cultivated) in the field and 67 plants in the common garden (33 wild and 34 cultivated) were collected (*n* = 321) in a single day. These were taken to the laboratory to measure herbivory (the area of the leaf eaten by herbivores) for each leaf with a leaf area meter (CID Biosciences Inc. CI-202, Camas, WA, USA).

### Direct physical defences

Using the same sample of leaves described above (*n* = 321), leaf toughness was measured as the force (N mm^−2^) needed to penetrate the leaf lamina with a handcrafted penetrometer. Leaf toughness was measured in the central part, avoiding the main ribs. All of the trichomes on the main rib and on the leaf border were counted. These areas of the leaf were selected because it has been reported that herbivores preferentially start their attack there. Since structural defences may jointly reduce leaf palatability, a cafeteria experiment was conducted. For this, leaf portions of similar size (8–16 cm^2^) from cultivated and wild plants were offered to the snail *Helix aspersa*. This snail is an effective instrument for measuring palatability because as a generalist herbivore it is not adapted to the specific defences of chaya. This was a choice experiment in which two leaf portions (one from wild and one from cultivated plants) were offered to a single snail which was placed, with the two leaf portions, in a 1-L plastic container. Snails were fasted for 48 h and weighed immediately before the experiment began. The containers were kept in a controlled environment chamber (Binder KBW 240, Tuttlingen, Germany) with a 16 h dark and 8 h light photoperiod at 26 °C. The area eaten and the weight of snails were recorded every 12 h for 2 days. Sample size was 60 experimental units (containers): 30 leaf portions from wild plants and 30 portions from cultivated plants, 50 % of which came from the field and 50 % of which were from the common garden, to see if the environment where the plant was grown affected leaf palatability.

### Indirect defences

During the survey of herbivores and in the same sample (see the *Herbivores and herbivory* subsection), we searched the entire plant for lepidopteran larvae. All caterpillars found were kept in a controlled environment chamber at 26 °C with a photoperiod of 12 h dark and 12 h light until either the adult or a parasitoid emerged. During captivity, the larvae were fed leaves from the same variety of chaya from which the caterpillar had been originally collected. Additionally, ants seen on the nectaries and petioles of five randomly selected leaves per plant for a random subsample of 23 cultivated (16 in the field and 7 in the common garden) and 20 wild plants (13 in the field and 7 in the common garden) were counted (*n* = 43).

### Data analysis

#### Herbivores and herbivory.

To assess the effect of domestication (a two-level factor: wild vs. cultivated plants) and the environment (a two-level factor: field vs. common garden), as well as their interaction on cumulative morphospecies richness and herbivore abundance for all sampling days, two generalized linear models (GLMs) with a Poisson (morphospecies richness) and quasi-Poisson (herbivore abundance) error distribution were fitted. Morphospecies were used instead of species because it was not possible to identify a large number of the insects to the species level. However, morphospecies are considered a reliable surrogate for species richness in invertebrates (Oliver and Beattie 1996). Leaf herbivory was expressed as a proportion of the whole leaf area and fitted to a linear mixed-effects model (LME) with domestication, the environment and their interaction as fixed factors and the plant as a random factor. Leaf herbivory was arcsine square-root transformed to improve normality.

#### Direct physical defences.

The effects of domestication, the environment and their interaction on per-plant direct defences (i.e. measurement from different leaves of the same plant was averaged) were assessed with GLMs (three models) with a Gaussian error distribution, except for the number of trichomes on the main rib, for which a Poisson error distribution was used. Because leaf perimeter (*F*_1, 18_ = 0.084, *P* = 0.77) and the length of the main rib (*F*_1, 18_ = 0.44, *P* = 0.51) were not statistically different between varieties in a random subsample (*n* = 20), it was not necessary to correct the number of trichomes per longitudinal unit. The leaf area eaten by snails was fitted to an LME with domestication, the environment and their interaction as fixed effects. Also, to account for any correlation between domestication treatments, the container was included as a random factor. A GLM with a Gaussian error distribution was used to assess the effect of the environment, the initial weight of the snail, total leaf area eaten, the proportion of leaf area of wild relative to cultivated plant eaten, as well as second-order interactions on the change in the weight of the snails.

#### Indirect defences.

The frequency of all parasitized larvae detected during all sampling days was compared between wild and cultivated plants with a chi-squared test, which was also used to assess differences in the frequency of larvae in wild and cultivated plants between environments. The effects of domestication, the environment and the domestication × environment interaction on per-plant ant morphospecies richness and ant abundance were assessed with GLMs with quasi-Poisson and Poisson error distribution, respectively.

An examination of residuals suggests that model fit was generally satisfactory. *A posteriori* power analyses showed that statistical power was on average within in or near the optimum suggested for behavioural sciences (80 %) by Cohen (1988) for direct defences (domestication = 99 %, environment = 71 %). For indirect defences (domestication = 60 %, environment = 62 %), herbivores and herbivory (domestication = 50 %, environment = 60 %) power was lower, but greater than that reported in some areas of ecology (47; Jennions and Møller 2003). All analyses were run in R software version 3.5.1. All data are available in **Supporting Information—Appendix S1**.

## Results

### Herbivores and herbivory

The richness of herbivore morphospecies per plant was 2.85 times greater in the field (5.02 ± 0.51) than in the common garden (1.76 ± 0.19; $\chi_{1}^{2}$ = 53.55, *P* < 0.01). However, the effect of domestication ($\chi_{1}^{2}$ = 0.19, *P* = 0.66) and the domestication × environment interaction ($\chi_{1}^{2}$ = 2.47, *P* = 0.18) were not statistically significant. Herbivore abundance per plant was 3.17 times greater on cultivated (58.47 ± 20.96) than on wild plants (18.42 ± 4.96) ($\chi_{1}^{2}$ = 784, *P* < 0.01), and 6.48 times more herbivores were found in the field (61 ± 18.21) than in the common garden (9.41 ± 2.37; $\chi_{1}^{2}$ = 457, *P* < 0.01). However, the domestication × environment interaction was not significant ($\chi_{1}^{2}$ = 316, *P* < 0.08).

**Figure 1.**
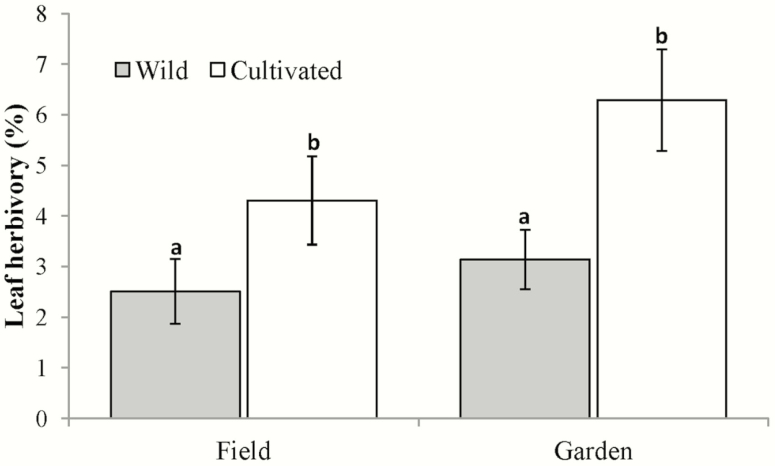
Leaf herbivory (% of the leaf eaten) in wild and cultivated *Cnidoscolus aconitifolius* in two different environments: in the field (field) and in a common garden (garden). Values are means ± 1 SE. Different letters indicate statistically significant differences.

Mean herbivory per leaf was 1.90 times greater for cultivated (5.53 ± 0.71 %) than for wild (2.91 ± 0.44 %) plants (*F*_1, 105_ = 7.81, *P* = 0.006). No significant effects of the environment (*F*_1, 105_ = 1.998, *P* = 0.160) or the domestication × environment interaction (*F*_1, 105_ = 0.091, *P* = 0.763) on herbivory were found (Fig. 1).

### Direct physical defences

**Figure 2.**
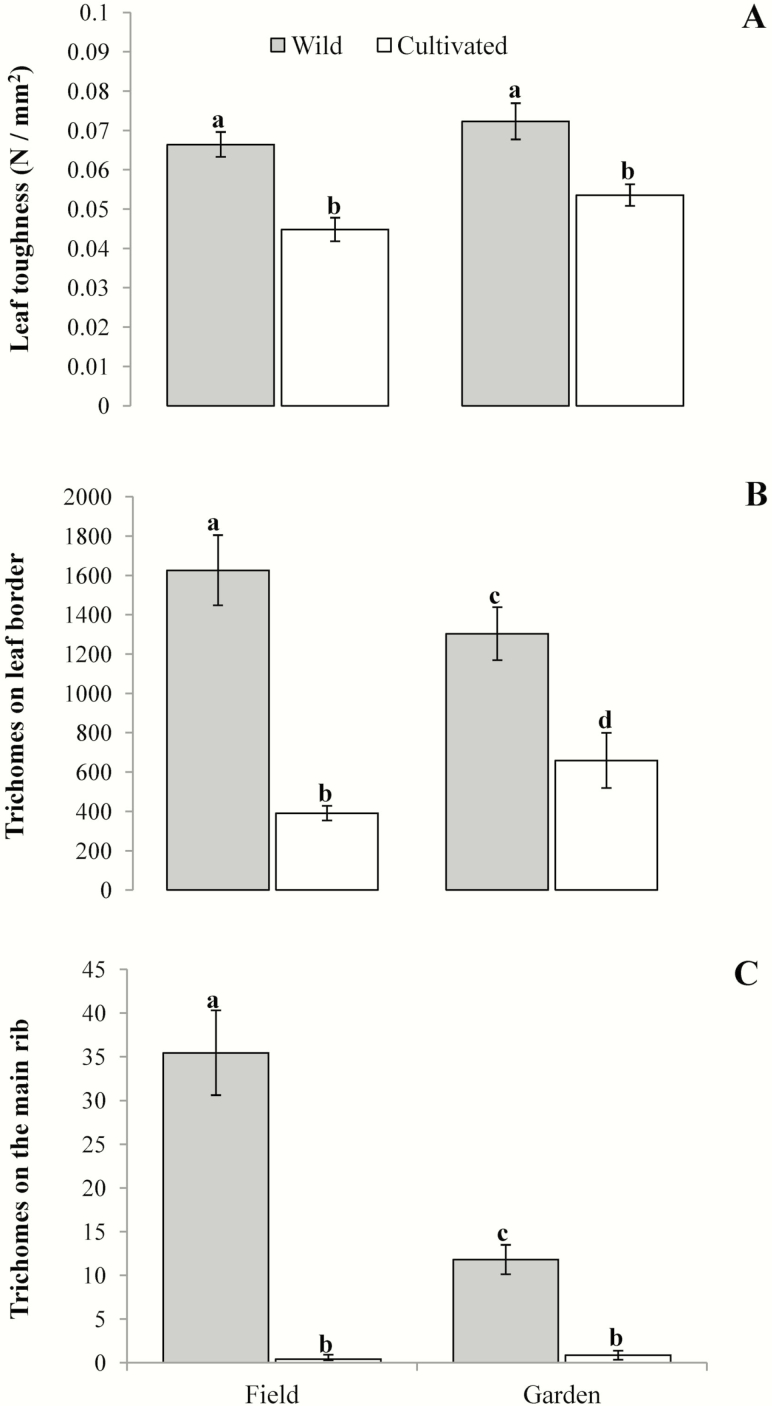
Leaf toughness (A), number of trichomes on leaf borders (B) and on the main rib (C) in wild and cultivated *Cnidoscolus aconitifolius* in two different environments: in the field (field) and in a common garden (garden). Values are means ± 1 SE. Different letters indicate statistically significant differences.

The leaves of wild plants (0.06 ± 0.01 N mm^2^) were 1.5 times tougher than the leaves of cultivated plants (0.04 ± 0.03 N mm^−2^) (*F*_1, 103_ = 27.94, *P* < 0.01). In contrast, the effects of the environment (*F*_1, 103_ = 3.54, *P* = 0.06) and of the domestication × environment interaction (*F*_1, 103_ = 0.14, *P* = 0.71) on leaf toughness were not significant (Fig. 2A). Wild plants had 2.55 % more trichomes on their leaf borders (1422.51 ± 108.54) than cultivated plants did (557.32 ± 89.54; *F*_1, 103_ = 38.503, *P* < 0.001). While environment did not affect the number of trichomes on the leaf border (*F*_1, 103_ = 0.04, *P* = 0.84), the domestication × environment interaction had a significant effect on this variable (*F*_1, 103_ = 4.19, *P* < 0.04). That is, wild plants had more trichomes on the leaf border in the field than in the common garden, while the opposite was observed in cultivated plants (field: wild = 1625.61 ± 178.48, cultivated = 390.65 ± 37.39; common garden: wild = 1303.03 ± 134.34, cultivated = 658.33 ± 139.92) (Fig. 2B). The number of trichomes on the main rib of the leaves was 30.22 times greater in wild (20.55 ± 2.59) than in cultivated plants (0.68 ± 0.32) ($\chi_{1}^{2}$ = 1248, *P* < 0.01). Also, plants had 2.79 times more trichomes on the main rib under field conditions (17.92 ± 3.69) than plants in the common garden did (6.41 ± 1.11) ($\chi_{1}^{2}$ = 305, *P* < 0.01). For this variable, the domestication × environment interaction was also statistically significant ($\chi_{1}^{2}$ = 26, *P* < 0.001). That is, while the number of trichomes on the main rib of the leaves was not significantly different between the two environments for cultivated plants (field = 0.59 ± 0.04, common garden = 2.93 ± 0.51), for wild plants the number of trichomes on the main rib of the leaves was 2.22 times greater in the field (21.74 ± 4.85) than in the common garden (9.81 ± 1.68) (Fig. 2C).

**Figure 3.**
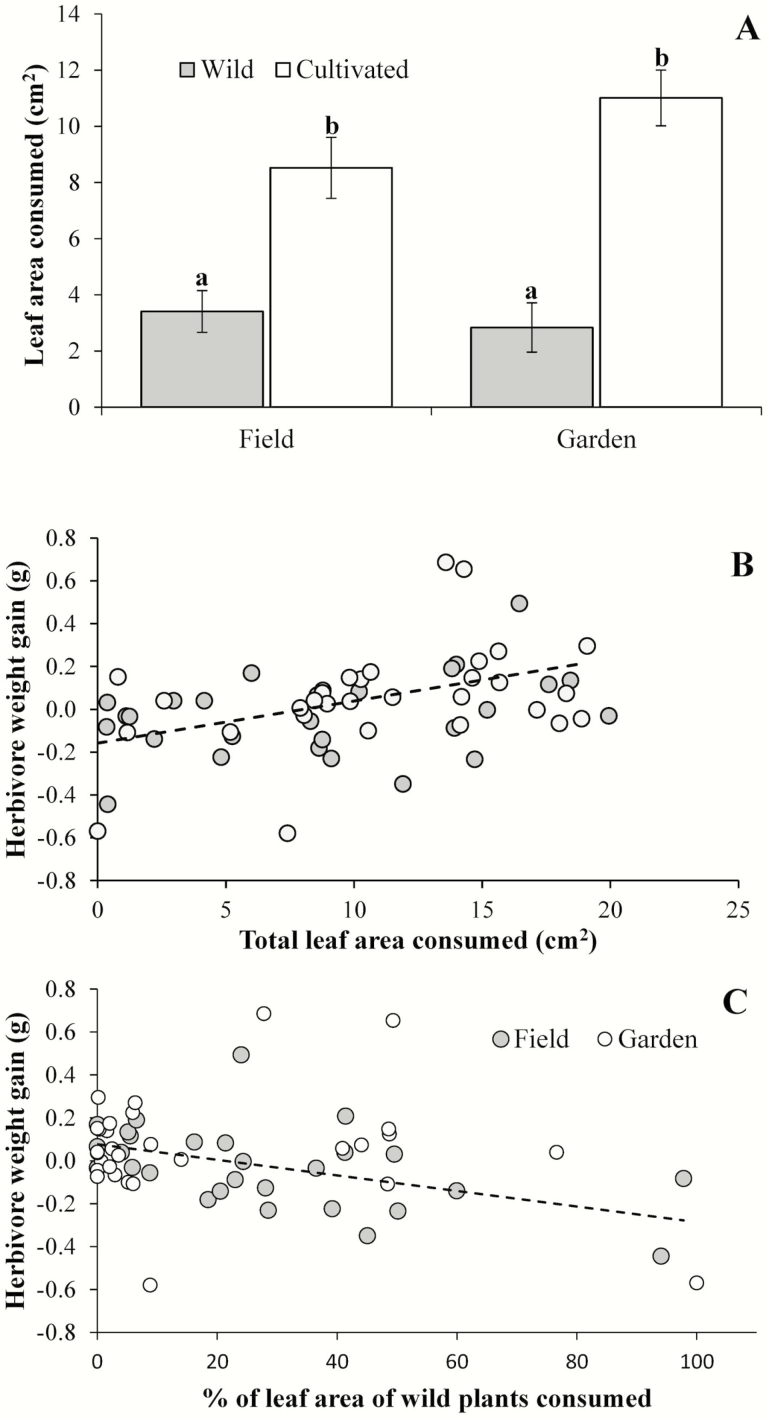
(A) Leaf area of *Cnidoscolus aconitifolius* consumed by the snail *Helix aspersa* in a cafeteria experiment. Leaf tissue from two plant varieties (wild and cultivated) grown in two different environments (field and garden) were offered to snails. Data in (A) are mean values ± 1 SE, different letters indicate statistically significant differences. Relationships of snail weight gain to total leaf area consumed (B) and the proportion of leaf area consumed of wild plants relative to the total area consumed (C). Grey and white circles in (B) and (C) represent plants grown in the field and in a common garden, respectively. The regression lines in (B) and (C) were calculated using the full data set (the slopes were statistically different from zero). Different letters in (A) indicate statistically significant differences.

Snails consumed 3.13 times more leaf area from cultivated (9.77 ± 0.75 cm^2^) than from wild plants (3.12 ± 0.57 cm^2^) (*F*_1, 58_ = 60.25, *P* < 0.01). The environment (*F*_1, 58_ = 0.91, *P =* 0.344) and the domestication × environment interaction did not significantly affect consumption by snails (*F*_1, 58_ = 3.19, *P =* 0.08) (Fig. 3A). The relationship between the weight gain by snails and total leaf area consumed was significant (*F*_1, 47_ = 6.16, *P* = 0.02) and positive (coefficient = 0.02 ± 0.01) (Fig. 3B). In contrast, the proportion of wild leaf area they consumed was also significant (*F*_1, 47_ = 5.94, *P* = 0.02) but negative (coefficient = −0.04 ± 0.02) (Fig. 3C). Explained variance was 11 % and 10 % for total area and the proportion of wild leaf area consumed, respectively. Neither the environment (*F*_1, 47_ = 1.24, *P* = 0.27) nor the initial weight of the snails (*F*_1, 47_ = 0.02, *P* = 0.89) had a significant effect. Finally, none of the second-order interactions were statistically significant (*F*_1, 47_ = 0.13–2.66, *P* > 0.12 in all cases).

### Indirect defences

In total, 25 lepidopteran larvae were seen and collected in the field: 13 on cultivated and 12 on wild plants. Parasitic wasps emerged from only two of the larvae collected from two cultivated plants. However, the frequency of parasitized larvae was not statistically different between cultivated and wild plants ($\chi_{1}^{2}$ = 0.46, *P* = 0.49). In the common garden, 11 larvae were seen and collected from cultivated and seven from wild plants, but none were parasitized. The number of larvae was not statistically different between environments ($\chi_{1}^{2}$ = 0.08, *P* = 0.77).

**Figure 4.**
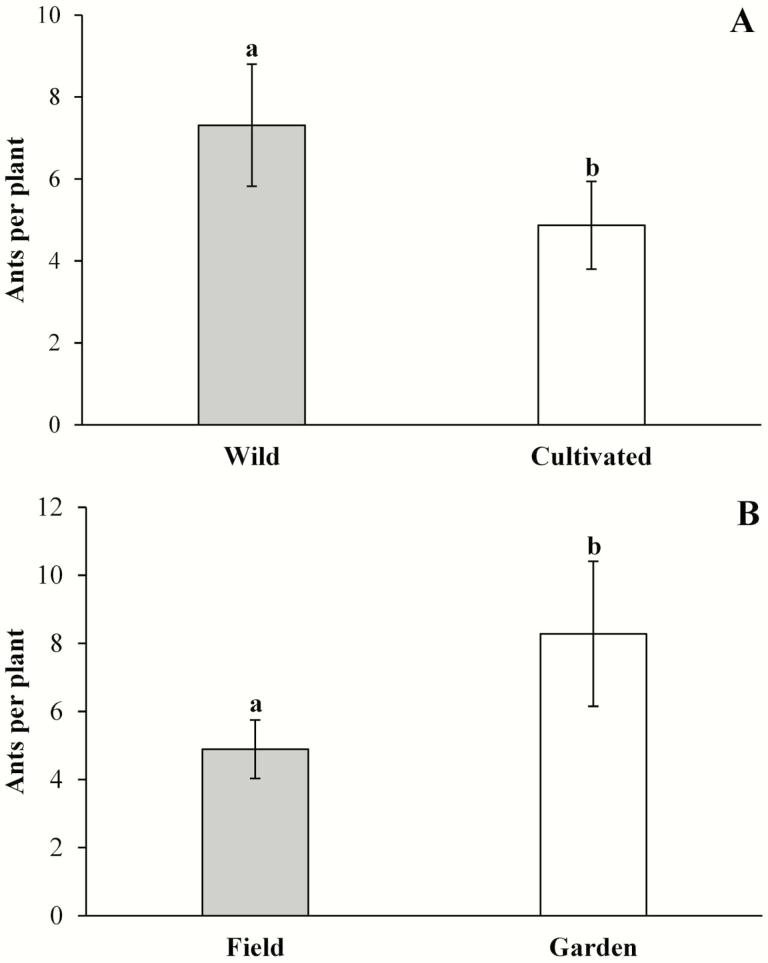
Ant abundance visiting extrafloral nectaries in wild and cultivated *Cnidoscolus aconitifolius* (A) in two different environments: in the field (field) and in a common garden (garden) (B). Data are mean values ± 1 SE. Different letters indicate statistically significant differences.

Morphospecies richness of ants on wild and cultivated plants was not statistically different ($\chi_{1}^{2}$ = 9.33, *P* = 0.92), and there was no difference between field and common garden plants ($\chi_{1}^{2}$ = 8.71, *P* = 0.43). Similarly, the domestication × environment interaction was not significant ($\chi_{1}^{2}$ = 8.64, *P* = 0.81). Ant abundance was 50 % greater (*F*_1, 39_ = 10.51, *P* < 0.01) on wild (7.31 ± 1.49) than on cultivated plants (4.87 ± 1.07) (Fig. 4A). Ant abundance was 69 % greater on plants in the common garden (8.28 ± 2.13) than in the field (4.89 ± 0.86) (*F*_1, 39_ = 10.51, *P* < 0.01) (Fig. 4B); however, the domestication × environmental interaction was not statistically significant (*F*_1, 39_ = 1.08, *P* = 0.29).

## Discussion

In this study we assessed the interplay of domestication *per se* and environmental effects on the herbivory, and direct and indirect anti-herbivory defences of a crop species (*C. aconitifolius*) in its domestication centre. Our results suggest that, as predicted, domestication increases herbivory while reducing direct and some indirect defences (ants). Environmental effects were also detected on some direct (trichomes) and indirect (ants) defences, suggesting that some anti-herbivore defences, in addition to be affected by domestication, are influenced by the environment. In fact, we also detected an interaction effect of domestication and the environment for trichome number. In general, our study suggests that not only does domestication *per se* affect anti-herbivore defences, but also that the environment may have some influence and sometimes interact with domestication. This generates a complex scenario in terms of anti-herbivory defences in plants under domestication.

As reported in a previous meta-analysis of the effect of domestication on resistance to herbivores (Whitehead *et al.* 2017), we found greater herbivore abundance and leaf herbivory in cultivated than in wild chaya. This was probably because cultivated plants had fewer trichomes and were not as tough as wild plants, characteristics that probably reduce the palatability and digestibility of plant tissues and thereby, reduce herbivore damage (War *et al.* 2012). This notion is also reinforced by the results of the cafeteria experiment in which, as expected, snails that ate more tissue gained more weight. In contrast, however, snails that ate more tissue from wild than from cultivated plants lost some weight (Fig. 3C). Despite the differences observed between wild and cultivated plants, both plant varieties exhibited relatively low levels of leaf herbivory (2–5 %), perhaps because wild and cultivated plants have a similar content of chemical defences such as hydrocyanic glycosides (Kuti and Kuti 1999) and condensed tannins (M. A. Munguía-Rosas, unpubl. data).

In contrast to the meta-analysis of Whitehead *et al.* (2017), where no consistent differences in anti-herbivore defences were found, our results match the domestication-reduced defence hypothesis completely. Differences between our study and that of Whitehead *et al*. probably result from our careful selection of wild and cultivated species as well as the control of environmental variables, which likely reduced random variation and contributed to the detection of a significant effect. In fact, Whitehead *et al.* (2017) recognized that their analysis included studies with geographic and environmental biases, including the comparison of pairs of species (crop vs. wild relatives) exposed to a completely different herbivore community. Unfortunately, we do not know if the greater herbivory seen in the cultivated plants resulted from changes in herbivore identity, abundance or consumption rate because we did not measure herbivory on a per-group/species basis.

Environmental conditions also affected the community of herbivores. Herbivore richness and abundance were greater in the field than in the common garden. This was probably because of the higher diversity of feeding resources and refugia found in the field locations (home gardens and secondary forest) relative to the common garden where chaya was planted as a monoculture. This finding agrees with those of previous studies that reported a bigger and more diverse herbivore community in polycultures and secondary forest than in monocultures (e.g. Chen and Welter 2002; Campos-Navarrete *et al.* 2015). It is important to highlight that, even though the environment has some effect on herbivore abundance, this did not cancel out the strong effect of domestication. That is, more herbivores were found on cultivated than wild plants regardless of the environment.

The number of trichomes was also influenced by the environment: plants growing in the common garden had fewer trichomes than plants in the field did. Previous studies have shown that in more stressful environments with greater resource availability, trichome density is greater in wild chaya (Abdala-Roberts and Parra-Tabla 2005), and this may explain why plants in the common garden had fewer trichomes than those in the field. In contrast to field locations, in the common garden there was no interspecific competition or light limitation (chaya is shade-intolerant) and therefore, resources availability is greater in this environment. Interestingly, we found an interaction effect of the environment with domestication in terms of the number of trichomes; that is, while there was a dramatic increase in the number of trichomes of wild plants in the common garden relative to their habitat in the field, there was little or no between-environment variation in the cultivated plants. We suggest that domestication *per se* has constrained the plastic response of trichome production in cultivated chaya. It is known that some crops exhibit highly canalized phenotypes and this is likely the result of artificial selection (e.g. Gage *et al.* 2017).

We found exciting results regarding indirect defences: more ants were seen visiting the nectaries of wild than cultivated plants. Because the defensive behaviour of these ants has been observed in the study species, this greater abundance of ants may have contributed, to some extent, to the reduced herbivory observed in wild plants. To our knowledge, no previous study had addressed the effect of domestication on ants involved in crop defence; therefore, more studies are needed to assess the generalizability of this result. Ant abundance per plant was also affected by environmental conditions: ants were significantly more abundant in the common garden than in the field, but in both environments more ants visited the nectaries of wild plants. This is probably because more homogeneous resources were available in the common garden than in the field (Price *et al.* 1980). We did not find any effect of domestication or environmental conditions on the incidence of parasitism of the leaf herbivores of chaya; however, this was probably due to the overall low rate of parasitism observed during the study; therefore, further research is needed with a larger sample.

To conclude, domestication increases herbivore abundance and leaf herbivory while reducing direct (leaf toughness and trichomes) and indirect defences (ants). This pattern perfectly matches the plant domestication-reduced defence hypothesis. Our results also suggest that the environmental variables associated with the habitat of cultivated and wild plants explain some of the variability observed in the community of herbivores (richness and abundance) and the plants’ defences (trichomes and ants). More interestingly, domestication and environmental factors may interact and generate more complex scenarios in terms of anti-herbivory defences.

In this study we assessed environmental and domestication effects on herbivores and anti-herbivory defences in chaya over a relatively short period of time. Because some herbivores and plant defences are influenced by the environment and this varies over time, a longitudinal study is needed to assess whether the patterns depicted in this study are temporally variable. Our results cannot be extrapolated beyond the domestication centre of chaya because the species has an extensive distribution and occurs in a wide variety of environments. Because the statistical power associated with the analysis of herbivores and indirect defences was somewhat less than ideal, other significant effects might be detected by increasing sample size. Finally, studies on other crop species are needed to assess the degree to which the patterns detected in this study can be generalized.

## Supporting Information

The following additional information is available in the online version of this article—

**Appendix S1.** Data.

plaa023_suppl_Supplementary_Appendix_S1
